# Methyl 5-chloro-2-nitro­benzoate

**DOI:** 10.1107/S1600536811044072

**Published:** 2011-11-02

**Authors:** Yan-Shu Liang, Bing-Ni Liu, Mo Liu, Deng-Ke Liu

**Affiliations:** a Tianjin University of Commerce, Tianjin 300134, People’s Republic of China; b Tianjin Institute of Pharmaceutical Research, Tianjin, 300193, People’s Republic of China

## Abstract

In the title compound, C_8_H_6_ClNO_4_, the nitro and acet­oxy groups attached to the benzene ring at neighbouring positions are twisted from its plane by 29.4 (1) and 49.7 (1)°, respectively. In the crystal, weak C—H⋯O hydrogen bonds link mol­ecules into layers parallel to (101). The crystal packing exhibits short inter­molecular C⋯O distances of 2.925 (3) Å.

## Related literature

The title compound is an inter­mediate of the oral vasopressin V_2_-receptor antagonist tolvaptan. For applications of tolvaptan, see: Nemerovski & Hutchinson (2010 ▶). For the synthesis of the title compound, see: Kondo *et al.* (1999 ▶). For a related structure, see: Liu *et al.* (2008 ▶).
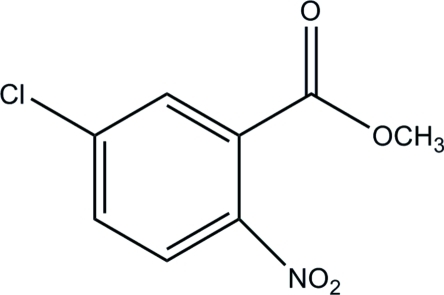


## Experimental

### 

#### Crystal data

C_8_H_6_ClNO_4_
*M* *_r_* = 215.59
Monoclinic, 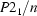

*a* = 4.2616 (9) Å
*b* = 22.470 (5) Å
*c* = 9.3894 (19) Å
β = 90.64 (3)°
*V* = 899.1 (3) Å^3^
*Z* = 4
Mo *K*α radiation
μ = 0.41 mm^−1^
*T* = 113 K
0.16 × 0.14 × 0.12 mm

#### Data collection

Rigaku Saturn diffractometer
Absorption correction: multi-scan (*CrystalClear*; Rigaku/MSC, 2005 ▶) *T*_min_ = 0.937, *T*_max_ = 0.952
5007 measured reflections
1564 independent reflections
1400 reflections with *I* > 2σ(*I*)
*R*_int_ = 0.031

#### Refinement

*R*[*F* ^2^ > 2σ(*F* ^2^)] = 0.041
*wR*(*F* ^2^) = 0.116
*S* = 1.07
1564 reflections
128 parameters
H-atom parameters constrained
Δρ_max_ = 0.27 e Å^−3^
Δρ_min_ = −0.27 e Å^−3^

### 

Data collection: *CrystalClear* (Rigaku/MSC, 2005 ▶); cell refinement: *CrystalClear*; data reduction: *CrystalClear*; program(s) used to solve structure: *SHELXS97* (Sheldrick, 2008 ▶); program(s) used to refine structure: *SHELXL97* (Sheldrick, 2008 ▶); molecular graphics: *SHELXTL* (Sheldrick, 2008 ▶); software used to prepare material for publication: *SHELXTL*.

## Supplementary Material

Crystal structure: contains datablock(s) I, global. DOI: 10.1107/S1600536811044072/cv5162sup1.cif

Structure factors: contains datablock(s) I. DOI: 10.1107/S1600536811044072/cv5162Isup2.hkl

Supplementary material file. DOI: 10.1107/S1600536811044072/cv5162Isup3.cdx

Supplementary material file. DOI: 10.1107/S1600536811044072/cv5162Isup4.cml

Additional supplementary materials:  crystallographic information; 3D view; checkCIF report

## Figures and Tables

**Table 1:** Hydrogen-bond geometry (Å, °)

*D*—H⋯*A*	*D*—H	H⋯*A*	*D*⋯*A*	*D*—H⋯*A*
C2—H2⋯O1^i^	0.93	2.53	3.206 (3)	130
C8—H8*B*⋯O3^ii^	0.96	2.47	3.200 (3)	132

Symmetry codes: (i) 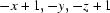; (ii) 
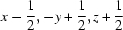
.

**Fig. 1.**
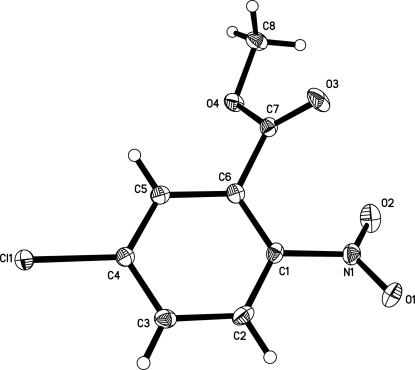
The molecular structure of (I), with the atom-numbering scheme and 50% probability displacement ellipsoids.

## supplementary crystallographic information

### Comment

Tolvaptan is an oral nonpeptide selective vasopressin V_2_-receptor antagonist indicated for the treatment of clinically relevant hypervolemic or euvolemic hyponatremia associated with heart failure, cirrhosis, or syndrome of inappropriate antidiuretic hormone (Nemerovski *et al.*, 2010). Now, we present the crystal structure of the title compound (I) (Kondo *et al.*, 1999), an intermediate of Tolvaptan.

In (I) (Fig. 1), all bond lengths and angles are normal and correspond to those observed in the analougs (Liu *et al.*, 2008). The acetoxy and nitro groups attached to the benzene ring at neighbouring positions are twisted from its plane at 49.7 (1) and 29.4 (1)°, respectively. In the crystal structure, weak C—H···O interactions (Table 1) link molecules into layers parallel to (101) plane. Short intermolecular C···O distances of 2.925 (3) Å observed in the structure.

### Experimental

To a stirred solution of the 5-chloro-2-nitrobenzoic acid (10 g, 50 mmol) in acetone(60 ml) was added K_2_CO_3_ (10.3 g, 74 mmol) and Me_2_SO_4_ (6.2 ml, 64.7 mmol). The mixture was heated at reflux for 30 min. The reaction mixture was then poured into an ice-water bath and extracted with ethyl acetate. The organic layer was separated and dried over MgSO_4_. then the filtrate was concentrate under reduced pressure to afford the methyl ester as a white solid. Pure compound (I) was obstained by crystallizing from methanol. Crystals of (I) suitable for X-ray diffraction were obstained by slow evaporation of an methanol solution.

### Refinement

All H atoms were positioned geometrically and refined using a riding model, with d(C—H) = 0.95 - 0.99 Å, and *U*_iso_(H) = 1.2 or 1.5 *U*_eq_.

### Crystal data

**Table:**

C_8_H_6_ClNO_4_	*F*(000) = 440
*M**_r_* = 215.59	*D*_x_ = 1.593 Mg m^−^^3^
Monoclinic, *P*2_1_/*n*	Mo *K*α radiation, λ = 0.71073 Å
Hall symbol: -P 2yn	Cell parameters from 2662 reflections
*a* = 4.2616 (9) Å	θ = 1.8–27.5°
*b* = 22.470 (5) Å	µ = 0.41 mm^−^^1^
*c* = 9.3894 (19) Å	*T* = 113 K
β = 90.64 (3)°	Prism, colorless
*V* = 899.1 (3) Å^3^	0.16 × 0.14 × 0.12 mm
*Z* = 4	

### Data collection

**Table:**

Rigaku Saturn diffractometer	1564 independent reflections
Radiation source: rotating anode	1400 reflections with *I* > 2σ(*I*)
confocal	*R*_int_ = 0.031
ω scans	θ_max_ = 25.0°, θ_min_ = 2.4°
Absorption correction: multi-scan (*CrystalClear*; Rigaku/MSC, 2005)	*h* = −4→5
*T*_min_ = 0.937, *T*_max_ = 0.952	*k* = −26→20
5007 measured reflections	*l* = −11→11

### Refinement

**Table:**

Refinement on *F*^2^	Primary atom site location: structure-invariant direct methods
Least-squares matrix: full	Secondary atom site location: difference Fourier map
*R*[*F*^2^ > 2σ(*F*^2^)] = 0.041	Hydrogen site location: inferred from neighbouring sites
*wR*(*F*^2^) = 0.116	H-atom parameters constrained
*S* = 1.07	*w* = 1/[σ^2^(*F*_o_^2^) + (0.0708*P*)^2^ + 0.3934*P*] where *P* = (*F*_o_^2^ + 2*F*_c_^2^)/3
1564 reflections	(Δ/σ)_max_ = 0.001
128 parameters	Δρ_max_ = 0.27 e Å^−^^3^
0 restraints	Δρ_min_ = −0.27 e Å^−^^3^

### Special details

**Table:**

Geometry. All e.s.d.'s (except the e.s.d. in the dihedral angle between two l.s. planes) are estimated using the full covariance matrix. The cell e.s.d.'s are taken into account individually in the estimation of e.s.d.'s in distances, angles and torsion angles; correlations between e.s.d.'s in cell parameters are only used when they are defined by crystal symmetry. An approximate (isotropic) treatment of cell e.s.d.'s is used for estimating e.s.d.'s involving l.s. planes.
Refinement. Refinement of *F*^2^ against ALL reflections. The weighted *R*-factor *wR* and goodness of fit *S* are based on *F*^2^, conventional *R*-factors *R* are based on *F*, with *F* set to zero for negative *F*^2^. The threshold expression of *F*^2^ > σ(*F*^2^) is used only for calculating *R*-factors(gt) *etc*. and is not relevant to the choice of reflections for refinement. *R*-factors based on *F*^2^ are statistically about twice as large as those based on *F*, and *R*- factors based on ALL data will be even larger.

### Fractional atomic coordinates and isotropic or equivalent isotropic displacement parameters (Å^2^)

**Table:**

	*x*	*y*	*z*	*U*_iso_*/*U*_eq_	
Cl1	0.83432 (12)	0.09111 (2)	1.09984 (5)	0.0272 (2)	
O1	0.1416 (4)	0.05968 (8)	0.42100 (17)	0.0423 (5)	
O2	−0.1876 (4)	0.12282 (9)	0.48951 (17)	0.0409 (5)	
O3	0.1575 (4)	0.22747 (7)	0.57507 (16)	0.0364 (4)	
O4	−0.0357 (3)	0.22861 (6)	0.78230 (15)	0.0263 (4)	
N1	0.0553 (5)	0.09256 (8)	0.51178 (19)	0.0282 (5)	
C1	0.2555 (5)	0.09452 (9)	0.6557 (2)	0.0224 (5)	
C2	0.4194 (5)	0.04329 (9)	0.7038 (2)	0.0273 (5)	
H2	0.4148	0.0092	0.6478	0.033*	
C3	0.6002 (5)	0.04214 (9)	0.8420 (2)	0.0263 (5)	
H3	0.7041	0.0077	0.8707	0.032*	
C4	0.6111 (5)	0.09270 (8)	0.9265 (2)	0.0215 (5)	
C5	0.4463 (5)	0.14420 (9)	0.8781 (2)	0.0224 (5)	
H5	0.4519	0.1783	0.9342	0.027*	
C6	0.2642 (5)	0.14566 (9)	0.7406 (2)	0.0218 (5)	
C7	0.1199 (5)	0.20438 (9)	0.6857 (2)	0.0228 (5)	
C8	−0.1662 (6)	0.28749 (10)	0.7466 (2)	0.0323 (5)	
H8A	0.0017	0.3157	0.7373	0.049*	
H8B	−0.3039	0.3001	0.8209	0.049*	
H8C	−0.2814	0.2850	0.6584	0.049*	

### Atomic displacement parameters (Å^2^)

**Table:**

	*U*^11^	*U*^22^	*U*^33^	*U*^12^	*U*^13^	*U*^23^
Cl1	0.0369 (4)	0.0243 (3)	0.0204 (3)	0.0011 (2)	−0.0024 (2)	0.00056 (18)
O1	0.0641 (13)	0.0388 (10)	0.0241 (9)	0.0017 (8)	0.0011 (8)	−0.0105 (7)
O2	0.0322 (10)	0.0596 (12)	0.0308 (9)	0.0044 (8)	0.0003 (7)	0.0013 (8)
O3	0.0481 (10)	0.0359 (9)	0.0256 (9)	0.0124 (7)	0.0201 (7)	0.0125 (7)
O4	0.0334 (9)	0.0244 (8)	0.0215 (8)	0.0032 (6)	0.0139 (6)	0.0031 (6)
N1	0.0335 (11)	0.0305 (10)	0.0206 (10)	−0.0064 (8)	0.0023 (8)	0.0002 (8)
C1	0.0231 (11)	0.0270 (11)	0.0174 (11)	−0.0047 (8)	0.0048 (8)	−0.0011 (8)
C2	0.0352 (12)	0.0223 (10)	0.0246 (11)	−0.0058 (9)	0.0087 (9)	−0.0063 (8)
C3	0.0331 (12)	0.0209 (10)	0.0252 (12)	−0.0008 (9)	0.0071 (9)	0.0024 (8)
C4	0.0255 (11)	0.0217 (10)	0.0176 (10)	−0.0033 (8)	0.0055 (8)	0.0018 (8)
C5	0.0280 (11)	0.0215 (10)	0.0178 (10)	−0.0035 (8)	0.0100 (8)	−0.0003 (8)
C6	0.0229 (11)	0.0252 (10)	0.0175 (10)	−0.0030 (8)	0.0099 (8)	0.0020 (8)
C7	0.0242 (10)	0.0258 (10)	0.0186 (11)	−0.0017 (8)	0.0049 (8)	−0.0004 (8)
C8	0.0416 (14)	0.0250 (11)	0.0307 (13)	0.0076 (10)	0.0123 (10)	0.0038 (9)

### Geometric parameters (Å, °)

**Table:**

Cl1—C4	1.876 (2)	C2—H2	0.9300
O1—N1	1.189 (2)	C3—C4	1.386 (3)
O2—N1	1.254 (3)	C3—H3	0.9300
O3—C7	1.174 (2)	C4—C5	1.425 (3)
O4—C7	1.254 (2)	C5—C6	1.500 (3)
O4—C8	1.472 (3)	C5—H5	0.9300
N1—C1	1.590 (3)	C6—C7	1.542 (3)
C1—C6	1.399 (3)	C8—H8A	0.9600
C1—C2	1.418 (3)	C8—H8B	0.9600
C2—C3	1.502 (3)	C8—H8C	0.9600
			
C7—O4—C8	115.29 (16)	C4—C5—C6	122.73 (18)
O1—N1—O2	118.6 (2)	C4—C5—H5	118.6
O1—N1—C1	117.30 (18)	C6—C5—H5	118.6
O2—N1—C1	124.10 (17)	C1—C6—C5	118.89 (18)
C6—C1—C2	118.4 (2)	C1—C6—C7	120.30 (18)
C6—C1—N1	121.16 (18)	C5—C6—C7	120.47 (17)
C2—C1—N1	120.40 (17)	O3—C7—O4	121.8 (2)
C1—C2—C3	122.38 (18)	O3—C7—C6	128.04 (18)
C1—C2—H2	118.8	O4—C7—C6	110.01 (16)
C3—C2—H2	118.8	O4—C8—H8A	109.5
C4—C3—C2	119.62 (19)	O4—C8—H8B	109.5
C4—C3—H3	120.2	H8A—C8—H8B	109.5
C2—C3—H3	120.2	O4—C8—H8C	109.5
C3—C4—C5	118.0 (2)	H8A—C8—H8C	109.5
C3—C4—Cl1	119.63 (16)	H8B—C8—H8C	109.5
C5—C4—Cl1	122.36 (15)		
			
O1—N1—C1—C6	152.58 (19)	N1—C1—C6—C5	177.54 (15)
O2—N1—C1—C6	−28.3 (3)	C2—C1—C6—C7	173.45 (17)
O1—N1—C1—C2	−30.1 (3)	N1—C1—C6—C7	−9.2 (3)
O2—N1—C1—C2	149.0 (2)	C4—C5—C6—C1	−0.1 (3)
C6—C1—C2—C3	0.1 (3)	C4—C5—C6—C7	−173.43 (18)
N1—C1—C2—C3	−177.31 (16)	C8—O4—C7—O3	0.0 (3)
C1—C2—C3—C4	−0.4 (3)	C8—O4—C7—C6	175.57 (17)
C2—C3—C4—C5	0.4 (3)	C1—C6—C7—O3	−48.6 (3)
C2—C3—C4—Cl1	179.53 (14)	C5—C6—C7—O3	124.6 (2)
C3—C4—C5—C6	−0.1 (3)	C1—C6—C7—O4	136.20 (19)
Cl1—C4—C5—C6	−179.27 (14)	C5—C6—C7—O4	−50.6 (2)
C2—C1—C6—C5	0.2 (3)		

### Hydrogen-bond geometry (Å, °)

**Table:**

*D*—H···*A*	*D*—H	H···*A*	*D*···*A*	*D*—H···*A*
C2—H2···O1^i^	0.93	2.53	3.206 (3)	130.
C8—H8B···O3^ii^	0.96	2.47	3.200 (3)	132.

Symmetry codes: (i) −*x*+1, −*y*, −*z*+1; (ii) *x*−1/2, −*y*+1/2, *z*+1/2.
